# *IRF4*-mediated Treg phenotype switching can aggravate hyperoxia-induced alveolar epithelial cell injury

**DOI:** 10.1186/s12890-024-02940-y

**Published:** 2024-03-15

**Authors:** He Langyue, Zhu Ying, Jiang Jianfeng, Zhu Yue, Yao Huici, Lu Hongyan

**Affiliations:** Department of Pediatrics, Affiliated Hospital of Jiangsu University, Zhenjiang, 212000 Jiangsu China

**Keywords:** Bronchopulmonary dysplasia, Regulatory T cells, Interferon regulatory factor 4, Forkhead transcription factor 3, Retinoic acid-related orphan nuclear receptor

## Abstract

Bronchopulmonary dysplasia (BPD) is characterized by alveolar dysplasia, and evidence indicates that interferon regulatory factor 4 (*IRF4*) is involved in the pathogenesis of various inflammatory lung diseases. Nonetheless, the significance and mechanism of *IRF4* in BPD remain unelucidated. Consequently, we established a mouse model of BPD through hyperoxia exposure, and ELISA was employed to measure interleukin-17 A (IL-17 A) and interleukin-6 (IL-6) expression levels in lung tissues. Western blotting was adopted to determine the expression of *IRF4*, surfactant protein C (SP-C), and podoplanin (T1α) in lung tissues. Flow cytometry was utilized for analyzing the percentages of FOXP3^+^ regulatory T cells (Tregs) and FOXP3^+^RORγt^+^ Tregs in CD4^+^ T cells in lung tissues to clarify the underlying mechanism. Our findings revealed that BPD mice exhibited disordered lung tissue structure, elevated *IRF4* expression, decreased SP-C and T1α expression, increased IL-17 A and IL-6 levels, reduced proportion of FOXP3^+^ Tregs, and increased proportion of FOXP3^+^RORγt^+^ Tregs. For the purpose of further elucidating the effect of *IRF4* on Treg phenotype switching induced by hyperoxia in lung tissues, we exposed neonatal mice with *IRF4* knockout to hyperoxia. These mice exhibited regular lung tissue structure, increased proportion of FOXP3^+^ Tregs, reduced proportion of FOXP3^+^RORγt^+^ Tregs, elevated SP-C and T1α expression, and decreased IL-17 A and IL-6 levels. In conclusion, our findings demonstrate that *IRF4*-mediated Treg phenotype switching in lung tissues exacerbates alveolar epithelial cell injury under hyperoxia exposure.

**Supplementary Information:**

The online version contains supplementary material available at 10.1186/s12890-024-02940-y.

 Bronchopulmonary dysplasia (BPD), characterized by abnormal development of alveoli and pulmonary vessels, is a chronic lung disease frequently observed in preterm infants necessitating oxygen therapy or mechanical ventilation. Regarding alveolar structure, the alveolar epithelium plays a crucial role in maintaining normal alveolar development [1]. Type II alveolar epithelial cells (AECIIs) are a type of lung progenitor cell involved in repairing lung epithelial injuries and differentiating into type I alveolar epithelial cells (AECIs, which are involved in gas exchange [2]. AECIIs, as the stem cells of the alveolar epithelium, exhibit a physiological state closely linked to lung injury occurrence and restoration of damaged alveolar epithelium morphology and function following hyperoxia exposure. Nonetheless, on the condition that exposed to numerous inflammatory factors, AECII structure becomes compromised, preventing its self-repair and hampering migration and differentiation into AECIs [3, 4]. Hyperoxia induces the activation of macrophages in immature lung tissues, resulting in the release of interleukin-6 (IL-6), interleukin-17 (IL-17), and regulatory chemokines [5]. These factors contribute to the degradation of elastic fibers of AECIIs, disrupt the homeostasis of AECIIs, and inhibit the growth and development of alveolar epithelial cells [5]. Furthermore, researchers have associated increased IL-6 and IL-17 and decreased IL-10 levels in neonates with a higher risk of future BPD [6, 7]. Growth factors and cytokines released by various immune cells form a complex and precise regulatory system, and yet the specific roles and mechanisms of each factor in maintaining or disrupting AECII homeostasis remain unclear.

Interferon regulatory factors (IRFs) are a class of active protein transcription factors produced by monocytes and lymphocytes with diverse functions, including participation in host immune responses, cell differentiation, and immune regulation [8, 9]. *IRF4*, a member of the IRF family, is critically involved in the differentiation and proliferation of immune cells and the activation of both innate and adaptive immune systems [10]. Additionally, *IRF4* regulates the onset and progression of chronic diseases, including allergic rhinitis, inflammatory bowel disease, and inflammatory arthritis [11–13]. *IRF4* regulates the release of IL-17 and IL-21 by controlling the transcriptional activity and accelerates the inflammatory process [14, 15]. Moreover, *IRF4* induces IL-10 production in acute lung infection by dendritic cells in lung tissue and aggravates inflammation of lung tissue [16]. *IRF4* has been determined to promote inflammatory response and aggravate inflammatory cell infiltration in asthma. *IRF4* has also been identified to be involved in the predisposing factors of COPD [17]. *IRF4* may interfere with pulmonary vascular development by affecting the proliferation of pulmonary vascular endothelial cells after hyperoxia exposure [18]. Nevertheless, the role of *IRF4* in BPD remains underexplored.

In recent years, *IRF4* extensively preoccupied with the mechanism of regulating the differentiation and development of T cells, B cells, as well as other immune cells. FOXP3^+^ regulatory T cells (Tregs) are a class of immune regulatory cells that secrete inhibitory factors including IL-10 and transforming growth factor-β to maintain immune homeostasis and tolerance. Forkhead box protein 3 (FOXP3) is the specific transcription factor for these cells [19]. In acute lung injury, FOXP3^+^ Tregs may directly affect AECII development and differentiation, thereby accelerating the resolution of inflammation [20]. Furthermore, Ravi et al. [21] linked a decreased number of FOXP3^+^ Tregs to BPD. Nevertheless, in certain microenvironments, including allergic asthma and rheumatoid arthritis, FOXP3^+^ Tregs can convert into other helper T cell subtypes, including Th17-, Th2-, and Th1-like Tregs [22, 23]. Among them, Th17-like Tregs co-express FOXP3 and retinoic acid-related orphan receptor γt (RORγt). These FOXP3^+^RORγt^+^Tregs can secrete pro-inflammatory cytokines including IL-17 and IL-21, thereby exacerbating the inflammatory response and contributing to airway mucosal injury [24]. Additionally, IRF4 has been identified as one of the transcription factors of Tregs and is downstream of the Treg-specific transcription pathway of FOXP3 [25, 26]. Moreover, *IRF4* can directly bind to the corresponding promoter and induce the expression of RORγt in T cells [27]. IRF4 is expressed in Th2-like Tregs, and its interaction with FOXP3 can affect the expression of target genes, promoting Th2-like Treg-mediated inhibition of Th2 anti-inflammatory responses [28]. Nevertheless, whether *IRF4* is involved in the conversion of FOXP3^+^ Tregs to FOXP3^+^RORγt^+^ Tregs remains uncertain.

Hence, we aimed to investigate whether IRF4 exacerbates alveolar epithelial cell injury in BPD mice by mediating the conversion of FOXP3^+^ Tregs to FOXP3^+^RORγt^+^ Tregs.

## Materials and methods

### Animals

Following a thorough review and approval by the Animal Ethics Committee of Jiangsu University (Grant no: UJS-IACUC-AP-2,020,030,304). C57BL/6 pregnant mice (gestational age 15–18 d) were procured from the Animal Center of Jiangsu University (Zhenjiang, China) under animal license number SCXK (Su) (2018-0008). *IRF4*-KO mice (Strain no. T012675), aged 10–14 weeks, were purchased from GemPharmatech (Nanjing, China) under animal license number SCXK (Su) (2018-0012). CRISPR/Cas9 technology was utilized to modify the IRF4 gene. All animals were housed under a 12-hour dark-light cycle in an appropriate environment and provided with adequate water and food.

### Establishment of experimental groups and models

C57BL/6 mice were randomly divided into two groups: normoxia and BPD groups. Mice in the BPD group were exposed to 85% oxygen in a self-made oxygen box, whereas the remaining mice were placed in atmospheric air and designated as the normoxia group [29, 30]. Besides, the mice were exposed to air or 85% oxygen from day 1 to day 14 and were euthanized at day 7 or day 14, respectively [31]. The oxygen concentration was maintained at 85% by monitoring with an oxygen meter, and the BPD mice were switched with those in the normoxia group every 12 h. The condition of the mothers and mice was monitored daily to prevent differences in maternal feeding caused by oxygen toxicity.

### Histological analysis

Respectively, five mice in the normoxia and BPD groups were euthanized on days 7 and 14 following air or hyperoxia exposure. The lung tissues were fixed and embedded in paraffin. Subsequently, 3 μm sections were prepared and stained with hematoxylin and eosin (HE). Furthermore, the structural and morphological changes in lung tissues were observed under a light microscope at 400× magnification. Radial alveolar counts (RACs) and mean linear intercept (MLI) were calculated in five slides from each specimen under a light microscope at 100× magnification, by senior pathologists. For each specimen, a vertical line was drawn from the center of the respiratory bronchiole to the distal pleura under a 100× light microscope, and the number of alveoli on the line was calculated as RAC [32]. Likewise, a cross line was drawn in the middle of the same field of view, the total length of the cross (L) and the number of alveolar septa (Ns) intersecting the cross were measured, and the MLI was calculated according to the formula MLI = L/Ns [33]. Three fields were selected from each section, and the average RAC and MLI were taken as one effective count.

### Measurement of IL-6 and IL-17 a protein levels using ELISA

Following exposure to air or hyperoxia, five mice from both the normoxia and BPD groups were euthanized on days 7 and 14, respectively. Lung tissues were taken and rinsed with normal saline to remove residual blood, and tissue homogenates were prepared using ultrasound. IL-6 and IL-17 A protein levels in the lung tissue homogenate were quantified utilizing IL-6(Vazyme, Nanjing, China) and IL-17 A (Lianke Biological Co., Ltd., China) ELISA kits, respectively. The total protein concentration in each sample was determined using a BCA protein detection kit, and the measurement of IL-6 and IL-17 A protein levels in lung tissues followed the manufacturer’s instructions. Each experiment was repeated five times.

### Western blot analysis of *IRF4*, SP-C, and T1α expression

Following exposure to air or hyperoxia, five mice in the normoxia and BPD groups were euthanized on days 7 and 14, respectively. Mouse lung tissues were lysed using RIPA (Beyotime, China) reagent containing protease inhibitors and PMSF (Beyotime, China). Besides, after repeated grinding with a glass homogenizer, the mixture was allowed to stand on ice for 30 min for adequate lysis. The mixture was centrifuged at 12,000 × *g* for 20 min, and the supernatant was collected as the total protein extract. Protein samples were mixed with loading buffer, boiled in hot water for 10 min, and subsequently cooled on ice [34]. The protein concentration was measured utilizing a BCA protein assay to ensure the total protein concentration of about 4ug/uL (Solarbio Science & Technology Co., Ltd., China). The extracted proteins were separated using SDS-PAGE (Vazyme Biotech Co., Ltd., China) and transferred to PVDF (Immobilon, USA) membranes via wet electrophoresis, and the membranes were blocked with 5% skim milk powder for one hour at room temperature (37 ℃). The following primary antibodies were added: mouse anti-β-actin antibody (cat.no. #3700, 1:1000, Cell Signaling Technology, USA), recombinant rabbit anti-*IRF4* monoclonal antibody (cat.no.#15,106, 1:500, Cell Signaling Technology, USA), rabbit anti-mouse SP-C polyclonal antibody (cat. no.90,716, 1:1000, Abcam, UK), and mouse anti-T1α monoclonal antibody (cat.no.sc-376,962, 1:500, Santa Cruz, USA). Incubation was conducted overnight at 4 ℃, and the membranes were washed three times with TBS containing 0.1% Tween 20 (TBST) at room temperature, and HRP-labeled goat anti-mouse IgG (cat.no.RS0001, 1:5000, Immunoway, USA) and goat anti-rabbit IgG (cat.no.RS0002, 1:5000, Immunoway, USA) were added. The membranes were incubated at room temperature (37 ℃) for one hour and rinsed with TBST three times. An enhanced chemiluminescence western blot kit (Vazyme Biotech Co., Ltd., China) was used for colorimetric detection, and Image J software was employed to analyze the grayscale values of the bands. Using β-actin as an internal reference, the expression levels of IRF4, SP-C, and T1α proteins were calculated relative. Each experiment was repeated five times, and the original blots were provided as supplementary information.

### Preparation of single-cell suspensions

Respectively, five mice in the normoxia and BPD groups were euthanized on days 7 and 14 after air or hyperoxia exposure. Fresh lung tissue was rinsed with normal saline to remove residual blood and ground on a 100 μm screen utilizing a 5 mL syringe piston handle. Moreover, the grinding process of lung tissue was kept in the environment of tissue diluent (Solarbio Science & Technology Co., Ltd., China). Single mouse cell suspensions were obtained after lysis of erythrocytes with ACK (Leagene, China). Subsequently, the single-cell suspension was carefully aspirated onto the separation liquid surface. Following a centrifugation of 900× g at room temperature for 40 min, the lymphocyte layer was carefully extracted into a tube for centrifugation. Cell washing solution (Solarbio Science & Technology Co., Ltd., China) was added, followed by centrifugation at 250 × *g* at room temperature for 10 min. The supernatant was discarded, and the cells were resuspended for further utilization [35, 36].

### Flow cytometry analysis of FOXP3^+^ Treg and FOXP3^+^RORγt^+^ Treg proportions

Surface staining was performed using PE-CD4 (cat. no.12-0043-82, Thermo Scientific, USA) and BV421-CD25 (cat. no.404-0251-82, Thermo Scientific, USA) mouse monoclonal antibodies. Subsequently, cells were fixed and permeabilized using FOXP3/Transcription Factor Staining Buffer Kit (Lianke Biological Co., Ltd., China). Following permeabilization, intracellular staining was performed employing APC-RORγt (cat. no.17-6981-82, Thermo Scientific, USA) and AF488-FOXP3 (cat. no.320,012, Thermo Scientific, USA) antibodies, according to the manufacturer’s protocols. Lymphocytes were gated in accordance with FCS-A and SSC-A properties. CD4^+^T cells were identified from the lymphocyte population. Tregs were identified from the CD4^+^ cell population. FOXP3^+^Tregs and FOXP3^+^RORγt^+^ (Th17-like) Tregs were analyzed separately. For the purpose of calculating the number of FOXP3^+^Tregs, FOXP3^+^Tregs were identified from the Tregs population by FOXP3 and CD25 [37]. To monitor the Tregs phenotype, FOXP3^+^RORγt^+^ (Th17-like) Tregs were identified from the Tregs population by CD25, FOXP3, and RORγt [38, 39]. Specifically, CD4^+^CD25^+^FOXP3^+^ cells were labeled as FOXP3^+^ Tregs, and CD4^+^CD25^+^FOXP3^+^RORγt^+^ cells were labeled as FOXP3^+^RORγt^+^ (Th17-like) Tregs. Flow cytometry analysis was conducted utilizing a CytoFlex flow cytometer (FACS Canto; BD, Franklin Lakes, NJ, USA), with a minimum of 1 × 10^5^ cells collected for each analysis, and each experiment was repeated five times.

### Construction and grouping of *IRF4*-KO mice

Studies were performed in *IRF4*-KO mice on a C57BL/6 background, purchased from GemPharmatech (Nanjing, China). The *IRF4* gene is located on chromosome 13 and consists of 3 transcripts. For the purpose of investigating the role of *IRF4* in BPD, CRISPR/Cas9 technology was employed to edit the *IRF4* gene. CRISPR/Cas9 technology efficiently knocks out protein-coding genes by deleting functional exons. We selected the IRF4-201 transcript (ENSMUST00000021784.9) as the knockout region. The targeting vector was designed to delete 421 base pairs from exon 3 to exon 5 of the IRF4 gene. Knocking out the region will result in the disruption of protein and the inability to perform its original function. The absence of the *IRF4* transcript in *IRF4*-KO mice was confirmed by Western blot analysis by used the recombinant IRF4 as positive control samples. Consequently, mature *IRF4*-KO homozygous mice were acquired. Deletion of this region results in the loss of protein function. *IRF4*-KO mice were cohoused at a male/female ratio of 3:1. Vaginal secretions of female mice were collected the next day, and mice with sperm detected in vaginal secretions through smear microscopy were considered pregnant. Furthermore, newborn *IRF4*-KO mice were exposed to 85% oxygen in a self-made oxygen box (KO-hyperoxia group) within two hours of birth, and the wild-type mice exposed to hyperoxia (WT-hyperoxia group) served as the control group. Both groups of mice were subjected to 85% oxygen for 14 days to ensure uniformity in the experimental conditions. In both groups, Surrogate mothers were exchanged every 12 h to prevent oxygen toxicity. Five mice in the WT-hyperoxia and KO-hyperoxia groups were euthanized on days 14 following hyperoxia exposure, respectively. No aphrodisiacal agents were employed during this experiment, and the fertility process was in line with the animal habits of the mice, and each experiment was repeated five times.

### Statistical analysis

GraphPad Prism 8.0.1 statistical software was employed to analyze the data, which were presented as mean ± SD. A t-test was employed to compare the two groups. The correlation between the two samples was analyzed by *Pearson* correlation. Statistical significance was set at *P* < 0.05, denoted by single asterisks (*), and *P* < 0.01, represented by double asterisks (**).

## Results

### Histopathological changes in lung tissues in the BPD mouse model

**Fig. 1:**
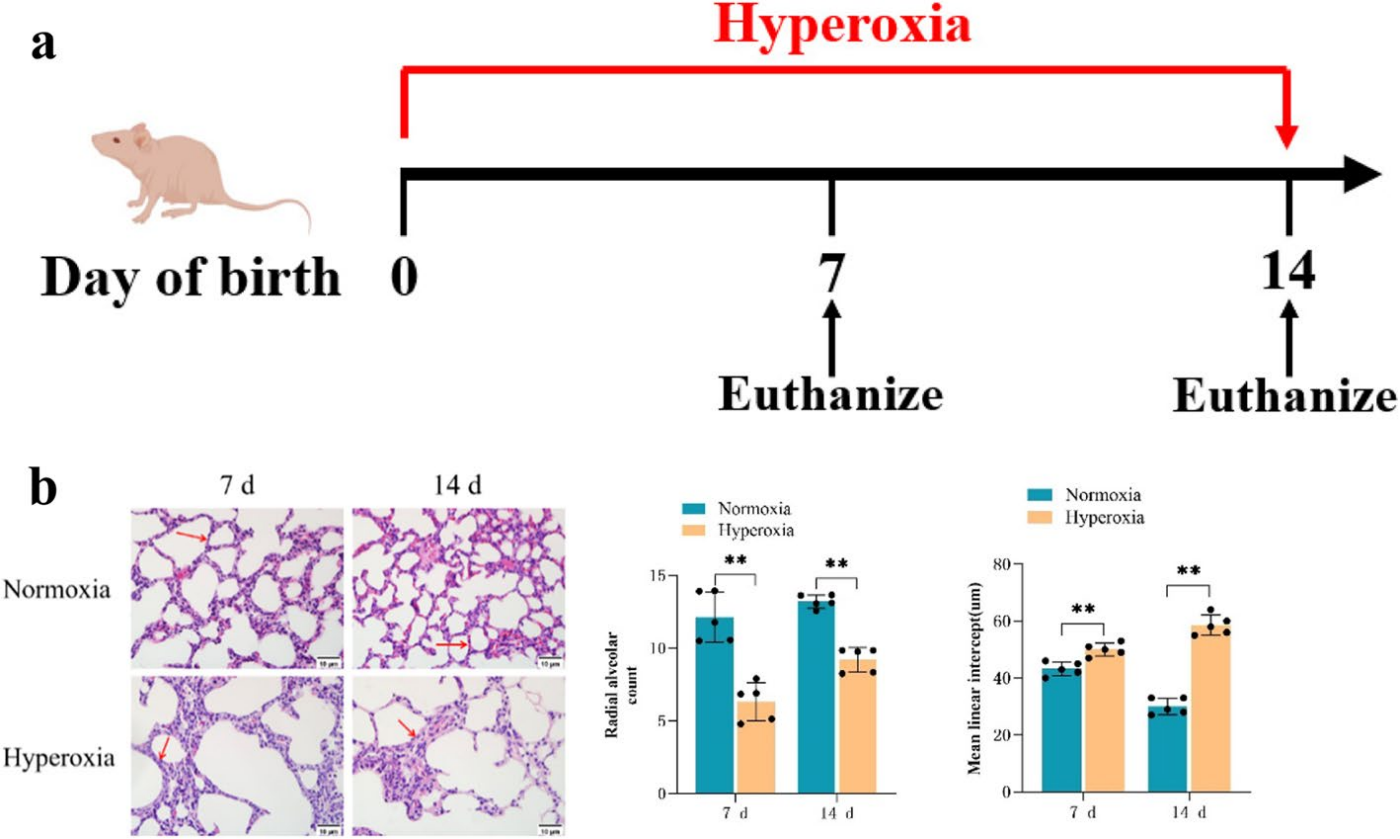
Analysis of lung histopathology and alveolar count in mice. **a** C57BL/6 mice were exposed to hyperoxia at birth and were sacrificed on days 7 and 14. **b** HE staining of lung tissues from mice in the air and hyperoxia groups (Scale bar = 10 μm; 400×), showed a signifcant decrease in radial alveolar count, and a signifcant increase in mean linear intercept. The representative data from five independent experiments. Data are presented as mean ± SD (*n* = 5, t-test one-way); *P < 0.05 and **P < 0.01, vs. normoxia group

We have effectively developed a murine model of BPD that is induced by hyperoxia (Fig. 1a). In the normoxia group, lung tissues exhibited normal morphology and structure, with well-developed and intact alveolar structures and a gradual increase in the number of alveoli. On an opposite, BPD mice demonstrated disrupted lung tissue structure and simplified alveolar structures. As these mice aged, the volume of their alveoli gradually increased, whereas the number of alveoli gradually decreased (Fig. 1b). Compared with the normoxia group mice, BPD mice demonstrated a substantial reduction in RACs and a significant increase in MLI simultaneously points (*P* < 0.05; Fig. 1b).

### Changes in cytokine levels and gene expression in BPD mouse lung tissues

**Fig. 2:**
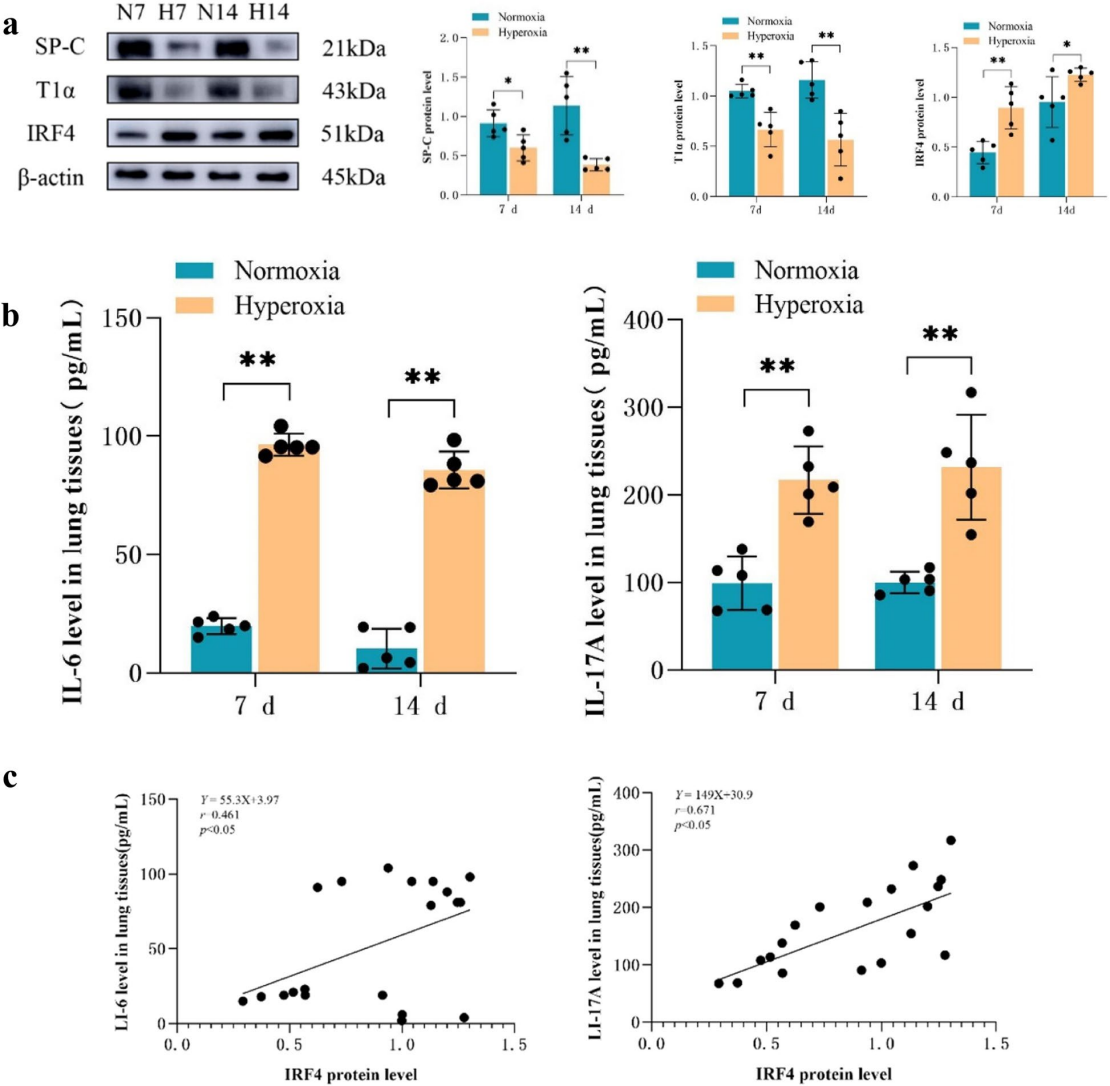
Abnormal transdifferentiation and inflammatory response induced by hyperoxia in mice. **a** Western blot analysis of SP-C, T1α, and *IRF4* expression in the mouse lung tissues of the normoxia and hyperoxia groups. β-actin was used as the loading control. **b** ELISA of IL-6 and IL-17 A expression in the mouse lung tissues of each group. **c** Correlation analysis of IRF4 with IL-6 and IL-17 A. The representative data from five independent experiments. Data are presented as mean ± SD (*n* = 5, t-test one-way); *P < 0.05 and **P < 0.01 vs. normoxia *g*roup

At the same time points, western blotting results indicated a significant decrease in the protein levels of SP-C (AECII-specific surface marker) and T1α (AECI-specific surface marker) in the lung tissues of BPD mice (*P* < 0.05; Fig. 2a). Concurrently, BPD mice’s lung tissues indicated a significant rise in IL-17 A and IL-6 compared to those of the normoxia group (*P* < 0.05; Fig. 2b). Furthermore, the expression of *IRF4* in the lung tissues of BPD mice was substantially increased simultaneously point compared with that in the normoxia group, which correlated with the trends observed in IL-6 and IL-17 A protein expression (*P* < 0.05; Fig. 2a, c).

### Altered proportions of FOXP3^+^ and FOXP3^+^RORγt^+^ Tregs

**Fig. 3:**
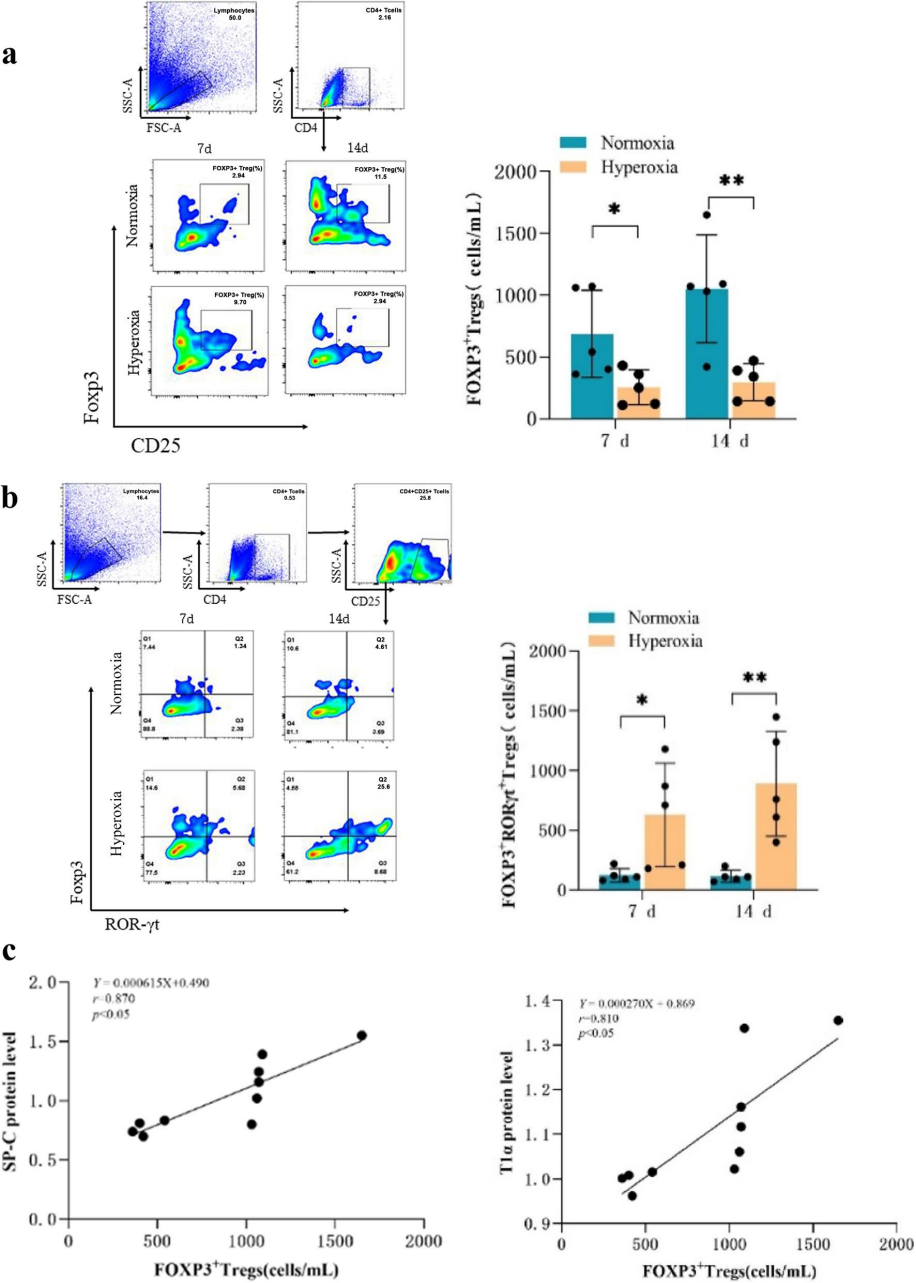
Hyperoxia-induced conversion of FOXP3 + Tregs to FOXP3 + RORγt + Tregs in mouse lung tissues. **a** Flow cytometry analysis of FOXP^+^ Tregs in the mouse lung tissues of the normoxia and hyperoxia groups. **b** Flow cytometry analysis of FOXP3^+^RORγt^+^ Tregs in the mouse lung tissues of the normoxia and hyperoxia groups. **c** Correlation analysis of FOXP3^+^ Tregs with SP-C and T1α. The representative data from five independent experiments. Data are presented as mean ± SD (*n* = 5, t-test one-way); *P < 0.0*5* and **P < 0.01 vs. normoxia group

Compared with that in the normoxia group, the proportion of FOXP3^+^ Tregs in the lung tissues of BPD mice gradually decreased at each time point (*P* < 0.05; Fig. 3a). Conversely, the proportion of FOXP3^+^RORγt^+^ Tregs in the lung tissues of BPD mice gradually increased in comparison to that in the normoxia group (*P* < 0.05; Fig. 3b). Additionally, with increasing postnatal age, the percentage of FOXP3^+^ Tregs in the lung tissues of normoxia group mice increased, which correlated with the trends observed in SP-C and T1α protein expression (*P* < 0.05; Fig. 3c).

### Effects of hyperoxia on Treg subpopulations in *IRF4*-KO mouse lungs

**Fig. 4:**
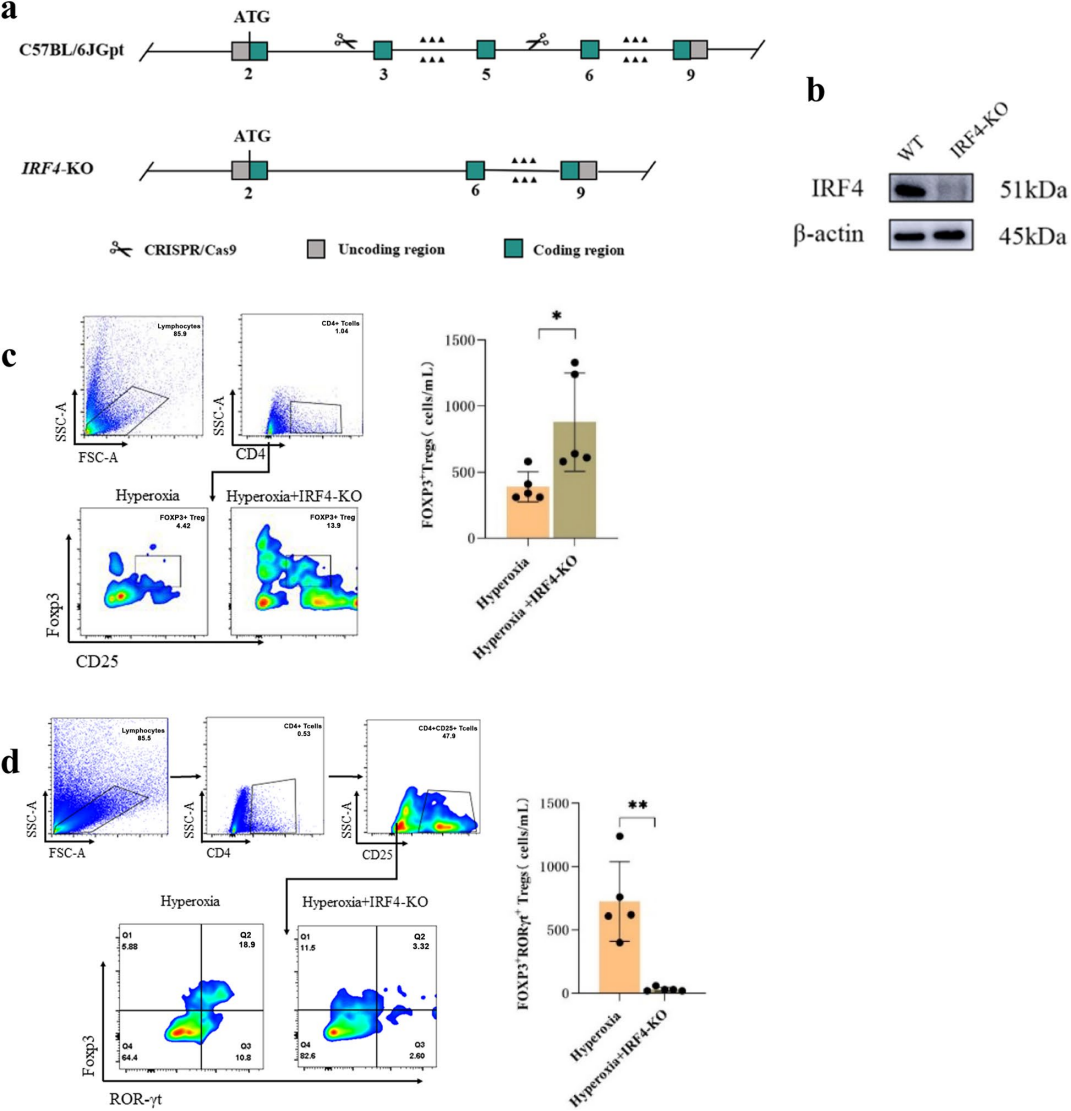
*IRF4* knockdown attenuates the conversion of FOXP3^+^ Tregs to FOXP3^+^RORγt^+^ Tregs in the lung tissues of mice after hyperoxia induction. **a** Schematic diagram of the construction of *IRF4*-KO mice by CRISPR/Cas9 technology. **b** Deletion of *IRF4* was confirmed by Western blotting (*n* = 5). **c** Flow cytometry analysis of FOXP3^+^ Tregs in the mouse lung tissues of WT-hyperoxia and KO-hyperoxia groups. **d** Flow cytometry analysis of FOXP3^+^RORγt^+^ Tregs in the mouse lung tissues of the WT-hyperoxia and KO-hyperoxia groups. The representative data from five independent experiments. Data are presented as mean ± SD (*n* = 5, t-test one-way); *P < 0.05 and **P < 0.01 vs. WT-hyperoxia group

To evaluate the effect of *IRF4* on FOXP3^+^Treg phenotype switching under hyperoxia, we established an *IRF4*-KO mouse model exposed to hyperoxia (Fig. 4a). Western blot indicated that the expression of IRF4 in IRF4-KO mice was substantially lower than that in WT mice, indicating high knockout efficiency (Fig. 4b). Flow cytometry results demonstrated that FOXP3^+^Treg proportion was higher (*P* < 0.05; Fig. 4c) and FOXP3^+^RORγt^+^ Treg proportion was lower (*P* < 0.05; Fig. 4d) in the KO-hyperoxia group than in the WT-hyperoxia group.

### Relief of lung injury and immunological changes in *IRF4*-KO mice

**Fig. 5:**
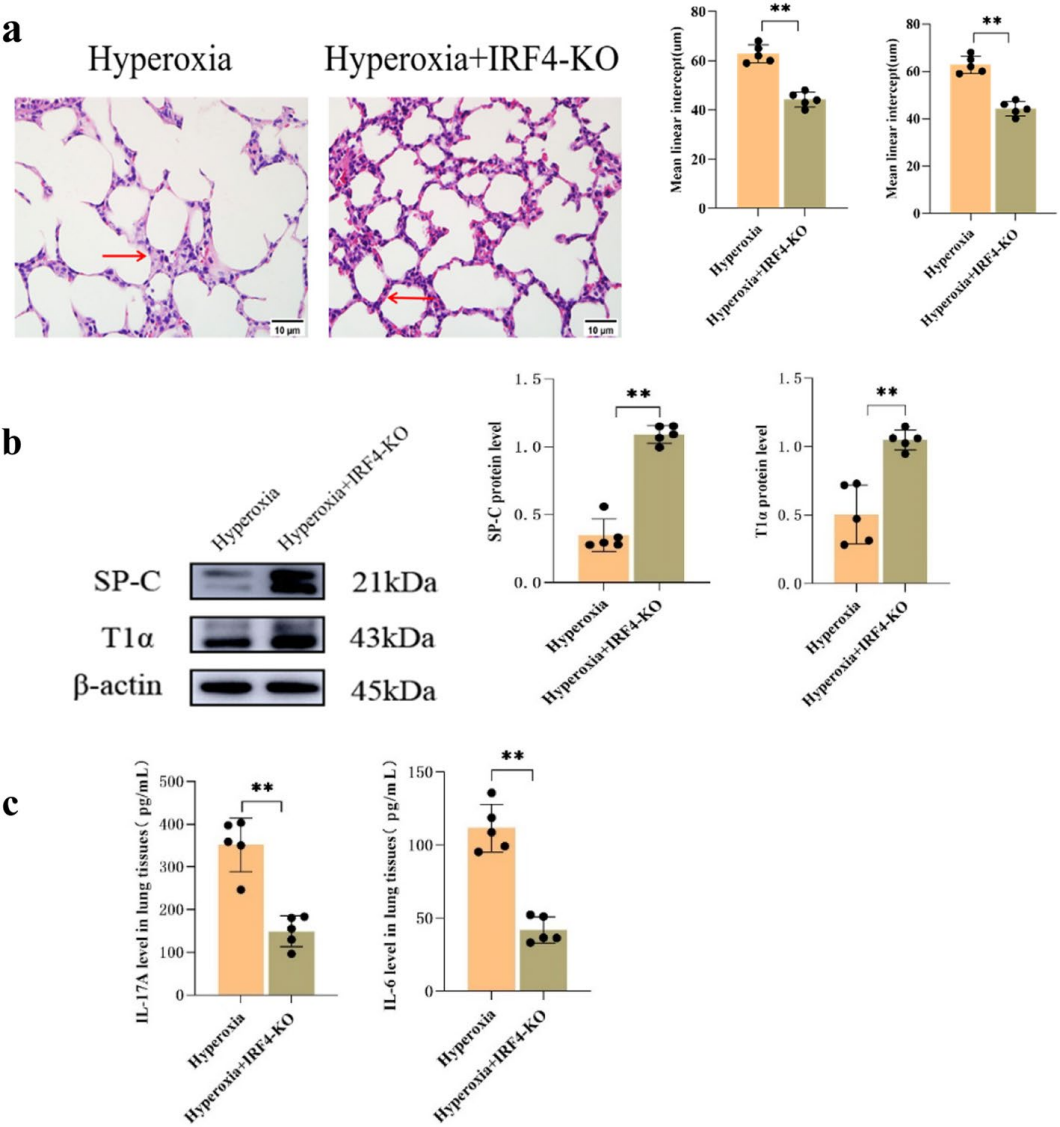
Improvement of hyperoxia-induced lung tissue injury by *IRF4* knockout. **a** HE staining of lung tissues from mice in the WT-hyperoxia and KO-hyperoxia groups (Scale bar = 10 μm; 400×), showed a significant increase in radial alveolar count, and a significant decrease in mean linear intercept. **b** Western blot analysis of SP-C and T1α protein expression levels in the lung tissues of WT-hyperoxia and KO-hyperoxia groups. **c** ELISA of IL-6 and IL-17 A expression in the mouse lung tissues of WT-hyperoxia and KO-hyperoxia groups. The representative data from five independent experiments. Data are presented as mean ± SD (*n* = 5, t-test one-way); *P < 0.05 and **P < 0.01 vs. WT-hyperoxia

Histological examination employing HE staining revealed that compared with the WT-hyperoxia group, the KO-hyperoxia group exhibited a well-preserved alveolar cavity structure and highly uniform alveolar volume (Fig. 5a). Western blotting results demonstrated increased protein expression of SP-C and T1α in the KO-hyperoxia group (*P* < 0.05; Fig. 5b). Furthermore, ELISA results indicated remarkably lower levels of IL-6 and IL-17 A in the lung tissues of the KO-hyperoxia group than in those of the WT-hyperoxia group (*P* < 0.05; Fig. 5c).

## Discussion

The primary pathological manifestation of BPD is alveolarization disorder, characterized by simple alveolar structure, thickening of the alveolar septum, as well as the proliferation of interstitial cells [40]. Hyperoxia exposure is among the primary etiologies of BPD. Miroslaw et al. [41] determined that hyperoxia exposure can result in altered genomic activity and elevated levels of oxidative stress in lung cells. Our previous studies found that long-term hyperoxia exposure also contributes to vascular endothelial growth factor and angiopoietin-1, resulting in impaired lung development [42]. Currently, neonatal mice exposed to high oxygen levels are a relatively mature animal model for BPD [29, 30]. In our study, we have established the BPD model of mice exposed to hyperoxia at 85% oxygen concentration, HE staining illustrated that the lung tissue structure of BPD mice was disordered, the alveolar volume was enlarged, and the structure was simple, showing BPD-like pathological manifestations, indicating that the model was effectively established. Mammalian alveolar epithelial cells are mainly composed of AECII and AECI. AECII can secrete various active proteins and trans-differentiation into AECI [43]. Surfactant protein C (SP-C) is a marker of AECII, and podoplanin (T1α) is a marker of AECI [35]. We determined that the expression levels of SP-C and T1α protein in the lung tissue of mice with BPD induced by hyperoxia were decreased, implying that alveolar epithelial cells were damaged and AECII trans-differentiation was abnormal. Long-term exposure to hyperoxia can induce a pro-inflammatory response in lung tissue, manifesting as the increase of various inflammatory factors and the apoptosis of alveolar epithelial cells [44, 45]. IL-17 and IL-6 are essential pro-inflammatory factors in the body, which can aggravate the inflammatory infiltration and injury of alveolar epithelial cells [46]. We determined that the levels of IL-17 A and IL-6 in the lung tissue of the hyperoxia group were remarkably increased, suggesting that the inflammatory response mediated by IL-17 A and IL-6 affected the process of AECII trans-differentiation and alveolarization.

In recent years, more and more scholars have paid attention to the role of the IRF family in lung diseases [47, 48]. In mammals, the IRF family consists of nine members (IRF1-IRF9), which mediates the transcription of interferon and play an essential role in cell differentiation and immune regulation [49]. IRF4, a member of the IRF family, is involved in the differentiation and proliferation of immune cells and plays a vital role in activating the innate and adaptive immune system [50]. IRF4 can induce airway inflammation in allergic rhinitis by participating in inflammatory responses involving Th2, Th9, and macrophages [11, 51]. Zhang et al. [52] determined that IRF4 can be involved in the polarization of alveolar macrophages, thereby inducing epithelial-mesenchymal transition and inducing the progression of pulmonary fibrosis. Nevertheless, the role of IRF4 in BPD remains ambiguous. Consequently, to investigate whether IRF4 plays a role in BPD, the expression of IRF4 protein was detected by Western blot. It was determined that the expression of IRF4 in the lung tissue of mice in the hyperoxia group increased, which was consistent with the trend of IL-17 A and IL-6, indicating that IRF4 may be related to the inflammatory response in BPD. To investigate further the function of IRF4 in BPD, we used CRISPR/Cas9 technology to knock out the *IRF4* gene in mice and performed hyperoxia exposure experiments. Western blot illustrated that the expression of IRF4 in IRF4-KO mice was substantially lower than that in WT mice, validating the deletion of the IRF4 gene. HE staining revealed that compared with the WT-hyperoxia group, the KO-hyperoxia group had improved pathological damage of lung tissue, more regular alveolar structure, and thinner alveolar septum. Western blot and ELISA results also indicated that the expression of SP-C and T1α was significantly increased, and the contents of IL-17 A and IL-6 were decreased. Those indicate that the inflammatory response of lung tissue was alleviated, and the damage of alveolar epithelial cells was enhanced after IRF4 knockout. Nonetheless, how *IRF4* is involved in aggravating the inflammatory response and alveolar damage remains unclear.

It has been determined that *IRF4* plays a crucial role in regulating the development and function of T lymphocytes and can regulate the differentiation of Treg [53]. Yu et al. [54] found that IRF4 affected the immunosuppressive function of Treg by regulating the conversion of FOXP3^+^Tregs to FOXP3^+^RORγt^+^Tregs, helping tumor cells to escape immune attacks. Several lines of evidence suggest that *IRF4* inhibits the expression of FOXP3 on Tregs and increases the expression of RORγt [25, 55]. When Tregs express both FOXP3 and RORγt, they are denoted as FOXP3^+^RORγt^+^Tregs [56]. FOXP3^+^RORγt^+^Tregs secrete IL-17, IL-21, and other characteristic factors of Th17 cells, exhibiting pro-inflammatory properties [57]. Massoud et al. [24] found a conversion of FOXP3^+^Tregs to FOXP3^+^RORγt^+^Tregs in asthma, which recruited inflammatory cell infiltration and aggravated airway hyperresponsiveness. Our study found that the number of FOXP3^+^Tregs in the lung tissue decreased in the hyperoxia group compared to the air group, while the proportion of FOXP3^+^RORγt^+^Tregs increased. This suggests a switching from FOXP3^+^Tregs to FOXP3^+^RORγt^+^Tregs in mice exposed to hyperoxia. It has been reported that a decrease in the population of FOXP3^+^Tregs and an increase in FOXP3^+^RORγt^+^Tregs exacerbate mucosal damage and airway inflammation [58]. BPD was associated with a reduction in FOXP3^+^Treg, leading to suppressive functional impairment and tilting the balance towards enhanced inflammation, which promotes the development of BPD in infants [21]. Our study identified a positive correlation between the reduction of FOXP3^+^Tregs and the decrease in SP-C and T1α expression. Conversely, our study found that the increase in FOXP3^+^RORγt^+^Tregs was consistent with the rising levels of IL-6 and IL-17 A. These findings suggest that the transformation of FOXP3^+^Tregs into FOXP3^+^RORγt^+^Tregs aggravates alveolar epithelial damage and contributes to the progression of lung inflammation in BPD. To further explore whether IRF4 regulates the conversion of FOXP3^+^Tregs to FOXP3^+^RORγt^+^Tregs in BPD, we used *IRF4*-KO mice for the next experiments. We found that the proportion of FOXP3 + RORγt + Tregs decreased in the KO-hyperoxia group, while the proportion of FOXP3 + Tregs increased. This suggests that knocking out *IRF4* inhibits the phenotypic switching of Tregs in the lung tissue of mice induced by hyperoxia. After exposure to hyperoxia, *IRF4*-KO mice exhibit a higher proportion of FOXP3^+^Treg as the predominant phenotype in lung tissue, leading to a more effective inhibitory function. FOXP3^+^Treg can promote the normal proliferation and differentiation of lung epithelial cells, inhibit lung inflammation, and resolve acute lung injury [17, 46]. It can be concluded that knocking out *IRF4* can inhibit the phenotype switching of Tregs in the lung tissue of mice induced by hyperoxia, thereby reducing inflammatory response and alveolar epithelial cell damage.

Nonetheless, there are still some limitations in this study. Despite the fact that the conversion of *IRF4* and Treg is mentioned several times in the manuscript, no in-depth conversion study has been conducted, and no in vitro and in vivo studies have been conducted to support the mechanism of how *IRF4* mediates transformation, which is based on literature support. Additionally, our data only demonstrated a decrease in AECII and AECI. Nonetheless, there is no direct evidence, including fluorescence colocalization experiments, to support the lack of AECII-to-AECI trans-differentiation except in our literature. In future experiments, we will also employ aerated fixation to assist the lung tissue closer to the viable state than formaldehyde fixation. Further investigations will be undertaken in the future to delve into these matters.

**Fig. 6:**
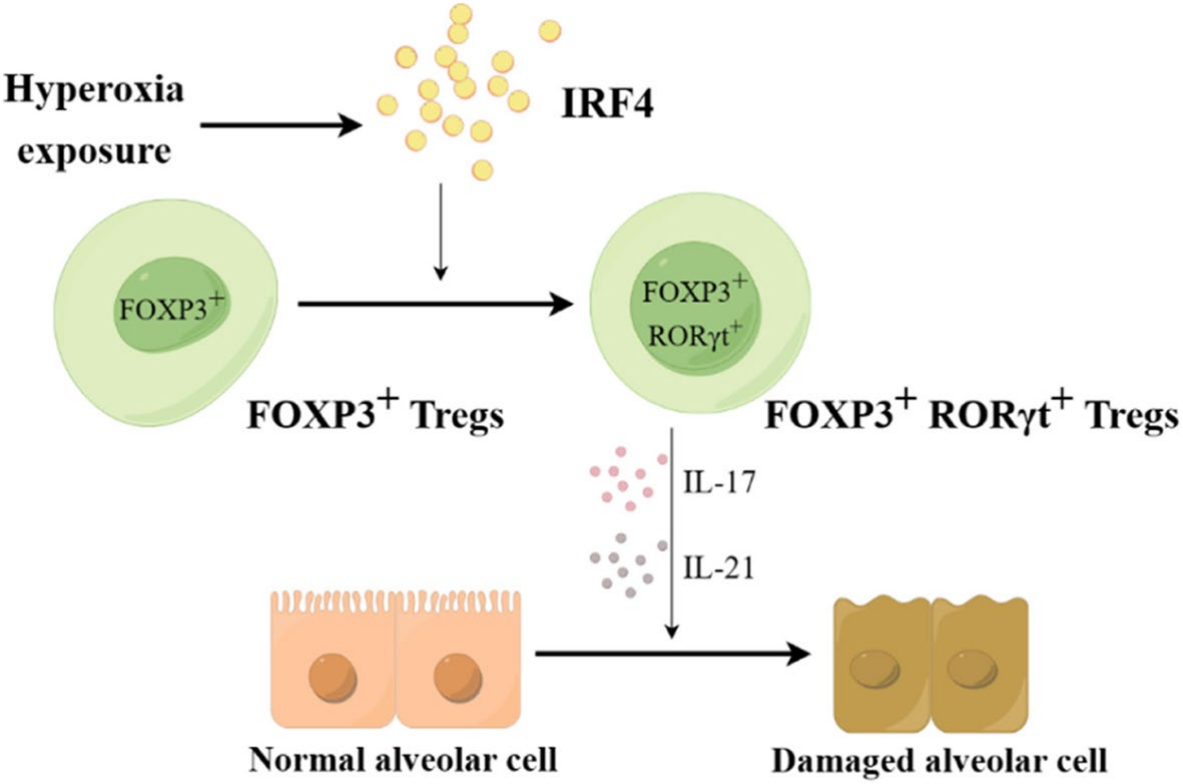
Graphical Abstract. Mechanism of *IRF4* regulation of hyperoxia-induced lung injury through conversion of FOXP3^+^ Tregs to FOXP3^+^RORγt^+^ Tregs under hyperoxia exposure

In conclusion, alveolar epithelial cell damage and alveolarization disorders may be associated with the regulatory effect of *IRF4* on Treg phenotype switching in mice with hyperoxia-induced BPD (Fig. 6). Knockout of *IRF4* can inhibit the FOXP3^+^Tregs phenotype to FOXP3^+^RORγt^+^Tregs phenotype, reduce lung tissue inflammation, and promote alveolar development, which may provide a new strategy for treating BPD.

### Supplementary Information

**Supplementary Material 1.**

## Data Availability

The data presented in this study are available on request from the corresponding author.

## Declarations

Animals from the National Institutes of Health (NIH) and ARRIVE.
