# Supplementary material for: IRF4-mediated Treg phenotype switching can aggravate hyperoxia-induced alveolar epithelial cell injury
Source: BMC Pulm Med. 2024 Mar 15;24:130. doi: 10.1186/s12890-024-02940-y (PMC10941512; doi:10.1186/s12890-024-02940-y)
Supplement: Supplementary file 1 — Supplementary Material 1. [file 12890_2024_2940_MOESM1_ESM.pdf]

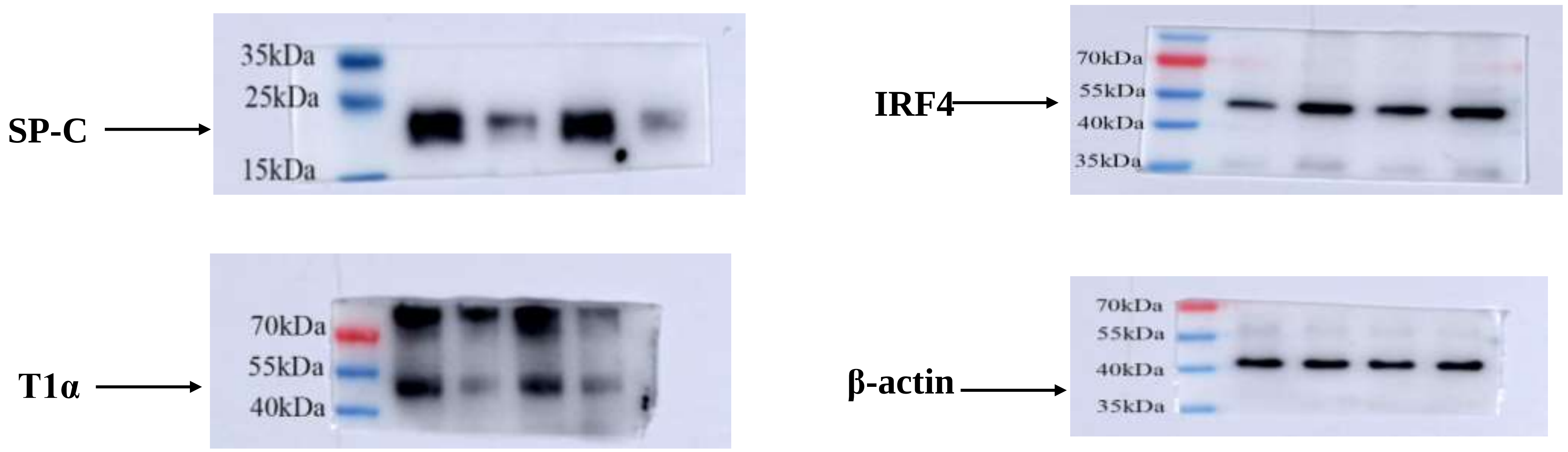

Figure 2 Abnormal transdifferentiation and inflammatory response induced by hyperoxia in mice. (a) Western blot analysis of SP-C, T1α, and *IRF4* expression in the mouse lung tissues of the normoxia and hyperoxia groups. β-actin was used as the loading control.

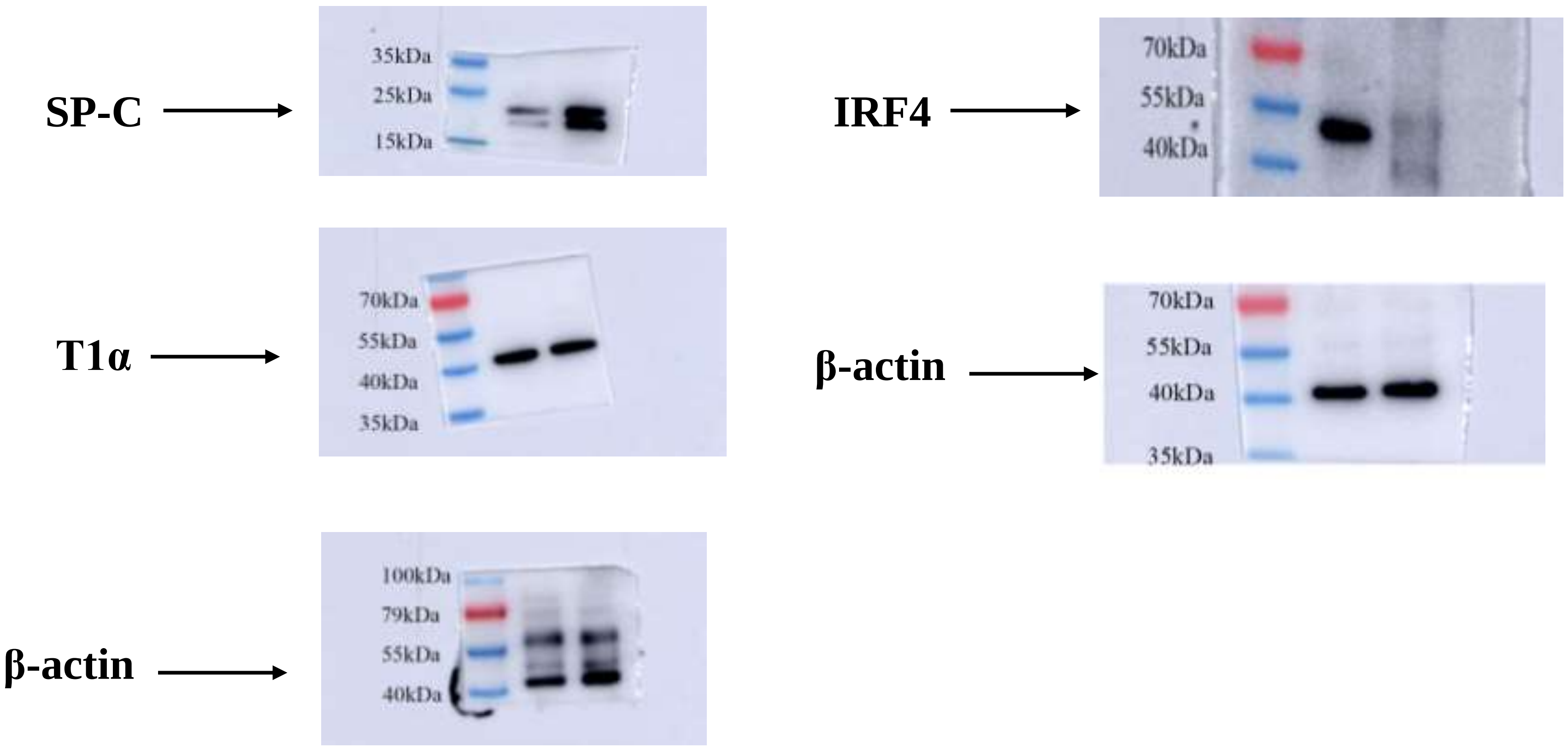

Figure 5 Improvement of hyperoxia-induced lung tissue injury by *IRF4* knockout. (b) Western blot analysis of SP-C and T1α protein expression levels in the lung tissues of WT-hyperoxia and KO-hyperoxia groups.

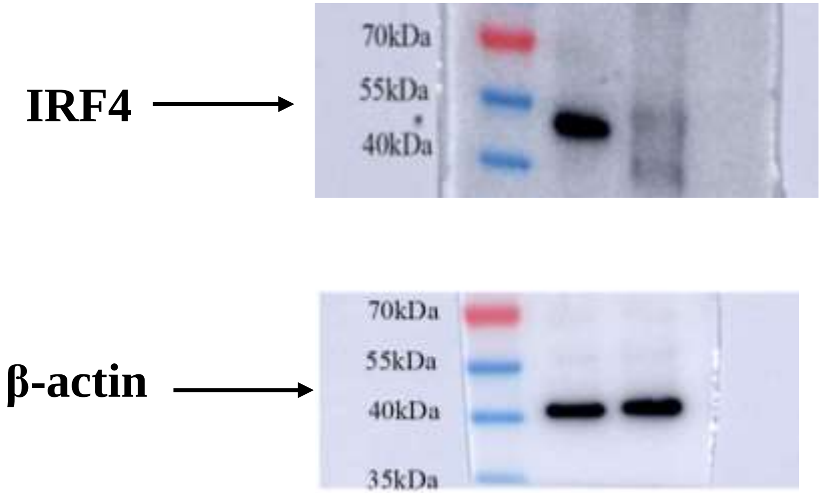

Figure 4b *IRF4* knockdown attenuates the conversion of FOXP3^{+} Tregs to FOXP3^{+}RORγt^{+} Tregs in the lung tissues of mice after hyperoxia induction. (b) Deletion of *IRF4* was confirmed by Western blotting.
